# Adherence to Antiretrovirals and HIV Viral Suppression Under COVID-19 Pandemic Interruption – Findings from a Randomized Clinical Trial Using Ingestible Sensors to Monitor Adherence

**DOI:** 10.1007/s10461-023-04118-9

**Published:** 2023-07-04

**Authors:** Yan Wang, Eric S. Daar, Yilan Huang, Di Xiong, Jie Shen, Linyu Zhou, Lisa Siqueiros, Mario Guerrero, Marc I. Rosen, Honghu Liu

**Affiliations:** 1 Section of Public and Population Health, University of California, Los Angeles (UCLA), Los Angeles, CA, USA; 2 Division of Infectious Diseases, David Geffen School of Medicine, UCLA, Los Angeles, CA, USA; 3 Department of Medicine, Division of HIV Medicine, Lundquist Institute at Harbor-UCLA Medical Center, Torrance, CA, USA; 4 Department of Biostatistics, Fielding School of Public Health, UCLA, Los Angeles, CA, USA; 5 School of Medicine, Yale University, New Haven, Connecticut, USA; 6 Department of Medicine, David Geffen School of Medicine, UCLA, Los Angeles, CA, USA

**Keywords:** The COVID-19 pandemic, Lockdown, HIV, Adherence, Viral load, Ingestible sensor

## Abstract

The COVID-19 pandemic had a significant impact on vulnerable populations, including people living with HIV. California implemented a coronavirus lockdown (stay-at-home order) in March 2020, which ended in January 2021. We evaluated the pandemic’s impact on both clinical outcomes of HIV RNA viral load (VL) and retention rate in a randomized clinical trial conducted from May 2018 to October 2020. The intervention group took co-encapsulated antiretrovirals (ARVs) with ingestible sensor (IS) pills from baseline through week 16. The IS system has the capacity to monitor adherence in real-time using a sensor patch, a mobile device, and supporting software. Both the IS and usual care (UC) groups were followed monthly for 28 weeks. Longitudinal mixed-effects models with random intercept and slope (RIAS) were used to fit log VL and self-reported adherence. The sample size of the study was 112 (54 in IS). Overall, the retention rate at week 28 was 86%, with 90% before the lockdown and 83% after the lockdown. The lockdown strengthened the associations between adherence and VL. Before the lockdown, a 10% increase in adherence was associated with a 0.2 unit decrease in log VL (β = −1.88, p = 0.004), while during the lockdown, the association was a 0.41-unit decrease (β = −2.27, p = 0.03). The pandemic did not have a significant impact on our adherence-focused intervention. Our findings regarding the intervention effect remain valid.

## Introduction

Globally, more than 37.7 million people were living with human immunodeficiency virus (HIV) infection and 27.5 million had access to antiretroviral (ARV) therapy (ART) at the end of 2020, with 1.5 million new infections diagnosed in 2021 [1]. The Undetectable = Untransmit-table (U = U) initiative further established the importance of maintaining high-level adherence and achievement of viral suppression [2]. The HIV care continuum is a public health model that outlined the five main steps from HIV diagnosis to viral suppression [3]. These steps include diagnosis, linkage to care, retention in care, adherence to antiretroviral therapy, and viral suppression. Successful navigation through the HIV continuum of care is associated with reduced morbidity and mortality and onward transmission of the virus [4]. Achieving a high percentage of coverage at each step of the continuum of care for people living with HIV is essential in a comprehensive strategy to address HIV [4]. Adherence to ART is required for viral suppression, prevention of the emergence of drug resistance, disease progression, and HIV transmission [5–7].

The coronavirus disease 2019 (COVID-19) pandemic raised new challenges to the HIV care continuum with periods in which much of population was under lockdown, curfews, and travel restrictions [8]. Maintaining each step in the HIV care continuum during the COVID-19 pandemic has been difficult from all perspectives, such as individuals accessing HIV testing and diagnostic facilities, initiating ART, refilling ARVs, traffic control, and suspended counselling services and prevention programs [8–10]. In-person clinic visits were widely affected by travel restrictions, clinic closures, and quarantine requirements. It has been highly recommended to incorporate telemedicine in high-resource settings to remedy the shocks to the healthcare system [10]. The COVID-19 pandemic also posed many challenges to clinical trials. Researchers were instructed to work remotely, and trials moved towards the home or online [11]. In some studies, the pandemic led to interruptions in the supply of investigational products and other clinical trial shipments [12]. With consideration of all the above, the integrity and interpretability before and during the COVID-19 pandemic should be taken into account when analyzing the intervention effects in clinical trials [13–17].

California issued a COVID-19-related lockdown on March 19, 2020, ordering all residents except first responders to stay home to prevent the spread of novel coronavirus (SARS-CoV-2). During this time, most of the HIV prevention programs were suspended and counseling services were stopped. Lundquist Institute at the Harbor-UCLA Medical Center remained open for the conduct of this ongoing clinical trial designed to address an innovative method for measuring and improving adherence to ART. While prioritizing the safety of the participants, the study continued for those already enrolled but stopped recruiting new participants during the lockdown. Despite efforts to maintain the integrity of the ongoing study, there remained concerns regarding the ability of participants to attend study visits. Lessons learned during the pandemic can be used to enhance patient care and the conduct of vital clinical trials. For the latter, additional consideration is needed to assess the impact of the pandemic on trial results.

This analysis was built upon several previously published papers. We evaluated the bioavailability of the co-encapsulated sensor pills with ARVs and confirmed that co-encapsulation does not affect the pharmacokinetics of eight commonly used ARV fixed-dose combination tablets: emtricitabine (FTC)/tenofovir disoproxil fumarate (TDF); FTC/tenofovir alafenamide (TAF); efavirenz (EFV)/FTC/TDF; abacavir (ABC)/lamivudine (3TC); dolutegravir (DTG)/ABC/3TC; rilpivirine (RPV)/TAF/FTC; elvitegravir (EVG)/cobicistat (COBI)/FTC/TAF; and bictegravir (BIC)/FTC/TAF [18]. We investigated the acceptability and satisfaction of the ingestible sensor system in a pilot study to ensure the tolerability of the larger co-encapsulated pill and systems, in which over 75% of respondents stated at each visit that the patch was very or somewhat comfortable [19]. We used qualitative methods to assess the perception of ingestible sensors of both participants and clinicians to understand the challenge and opportunities from both patients’ and providers’ perspectives [20]. We evaluated the accuracy, effectiveness, sustainability, as well as participant satisfaction with different aspects of the IS system in the main randomized clinical trial [21]. The IS system was well accepted by participants and its use was associated with improved adherence and lower HIV RNA viral load (VL). In this paper, we evaluated the impact of the COVID-19 pandemic on our main study, both clinical outcomes and retention rate.

## Methods

### Recruitment and Randomization

All participants were recruited from the Lundquist Institute at the Harbor-UCLA Medical Center (HUMC), and Long Beach Comprehensive Care HIV clinics from May 2018 to February 2020 (ClinicalTrials.gov Identifier: NCT02797262). The last follow-up study visit happened in October 2020. The inclusion criteria were (1) HIV-infected individuals in care; (2) at least 18 years old; (3) demonstrated ability to take co-encapsulated medication at the time of screening; (4) on ART with evidence of suboptimal adherence as defined by < 90% adherence over the last 28 days by self-report, or over the last 6 months as perceived by the treating clinician based on missed appointments, reported missed doses, or viral load elevations. The exclusion criteria were (1) inability to follow the study procedures manifested during the intake, as evidenced by mental confusion, disorganization, intoxication, withdrawal, risky or threatening behavior; (2) pregnancy. Enrolled individuals were assigned to either the ingestible sensor (IS) group or the usual care (UC) group by a stratified randomization procedure on (1) whether HIV RNA viral load was detectable and (2) whether the participants were on single or multiple pill regimen. This study was approved by UCLA and Lundquist Institute Institutional Review Board. Written consent forms were acquired from all study participants.

### Intervention - Ingestible Sensor System

**Fig. 1:**
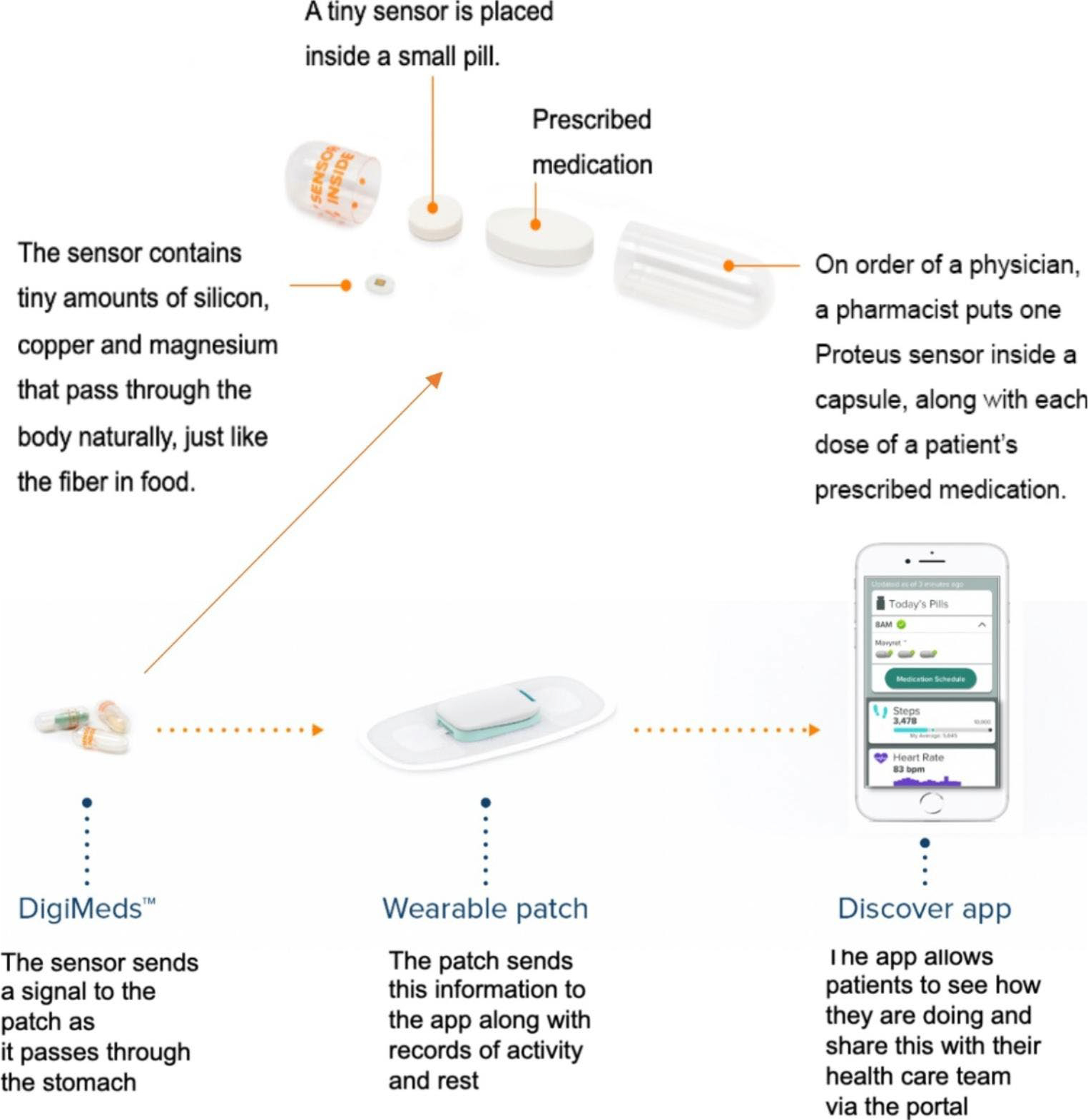
Ingestible sensor system with co-encapsulated medication with sensor pills

The intervention arm (IS group) used an ingestible sensor system that included co-encapsulated ingestible sensor with ARVs, a cutaneous patch with Bluetooth technology connected with a mini-iPad or iPhone that communicated with a server that was programmed to provide pre- and post-dose text messages if medication event was not detected (Fig. 1). After a dose is ingested, the sensor pill sent a signal to the patch (placed on the left upper quadrant of abdomen), in turn, the patch sent a signal to a personal device (either iPhone or iPad) through Bluetooth signal, and the personal device shared with the server. All participants followed for up for 28 weeks. The IS group used the ingestible sensor system for 16 weeks, followed by a 12-week post-intervention period to assess the sustainability of the system. The control arm was in usual care (UC group). Details of the IS system were described in previous studies [18–20]. The scheduled monthly study visits included a questionnaire at each visit and blood drawn at 4-, 8-, 12– 16- and 28-weeks.

### Measures

#### Missing Study Appointments

Our study records provided data on completed or missed appointments, or dropouts due to the pandemic or difficulties. We defined the retention rate as the percentage of participants who completed the last visit at week 28. The retention rates were calculated for all participants as well as before or after the lockdown. The missing monthly appointments (“no show” or “cancelled”) during the 28-week study period were profiled and compared between the two study arms.

#### IS Adherence

The daily ART adherence events for IS group was recorded by the IS system, with pre- and post- reminder messages if a missed medication event (i.e., ingestion) was detected. The IS-monitored rate of missed dose was defined as the proportion of participants in the IS group who missed the dose in a specific study day.

#### Self-reported Adherence

Self-reported ART adherence measures were assessed by questionnaires administrated monthly during study visits for both groups. We used weekly recall to summarize the ART adherence (“in the past seven days, how many doses did you miss?”), which considered both potential recall bias and variation in self-reported measures.

#### Plasma HIV RNA

Plasma HIV RNA VL was used as a biological outcome of adherence. During the intervention period (first 16 weeks), VL was measured monthly for both IS and UC arms. During the post-intervention period, VL was measured at week 28.

### Statistical Analysis

Participants were enrolled from June 2018 to February 2020. For a given calendar date, they were in different phases of the study. We have both study week and calendar week for each data point. Calendar week was defined as the number of weeks to/since the pandemic lockdown began, March 19, 2020 (ranged from − 93 to 13). Study week corresponded to every four-week appointment (ranged from 0 to 28). We included all data from June 11, 2018 to March 18, 2020 as the pre-lockdown group, and data from March 19, 2020 to October 15, 2020 as the post-lockdown group. Participants completing the 28-week visit before the lockdown were classified “before lockdown,” while those who had at least one study visit after the lockdown were classified as “after lockdown.” Baseline characteristics of study population were compared between the two subgroups within IS arm and UC arm. Two sample t-tests were used to compare continuous variables (age, HIV + years, years under ARV treatment, and baseline VL). Chi-Square test or Fisher exact test were used for categorical variables (gender, race/ethnicity, education, employment, AIDS history, and detectable VL at baseline). Linear mixed models with random intercept and slope (RIAS) were used to estimate the association between the HIV VL and the change in adherence. In the model, we used bimonthly average self-reported adherence, including covariates such as demographics, bimonthly average CD4 count, and other HIV-associated variables (multiple regimens, detectable VL at baseline, years under ARV treatment). The bimonthly average self-reported adherence was calculated as: average adherence at weeks 4 and 8; average adherence at weeks 12 and 16; adherence at week 28.

## Results

**Fig. 2:**
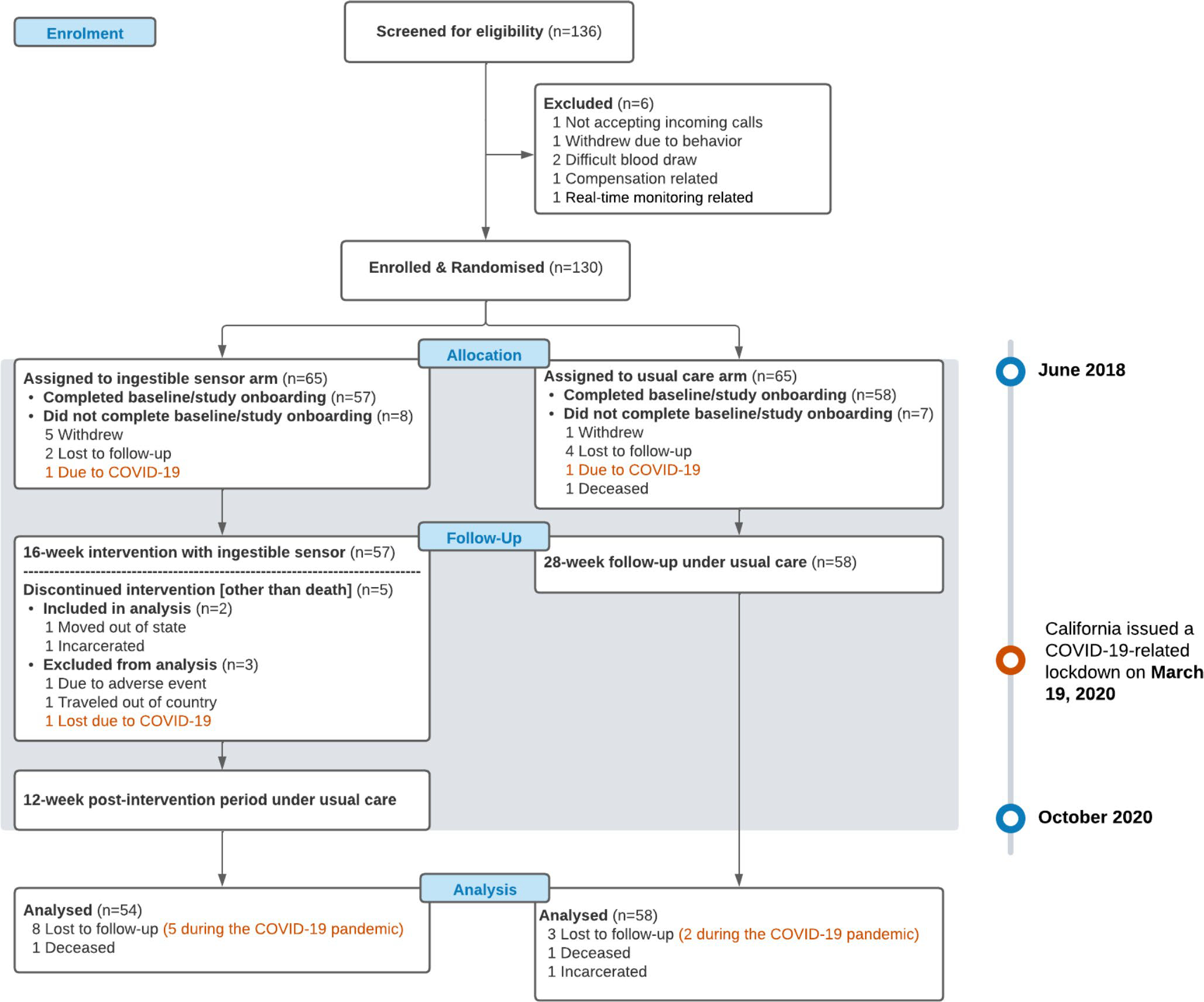
Recruitment and Randomization of Patients

The study screened 136 participants from 2018 to 2020 (Fig. 2). Six patients did not meet the inclusion criteria and 130 were randomized into the study. There were 18 patients excluded from the analysis; six withdrew from the study without providing reasons, six were lost to follow-up, 3 withdrew due to COVID-19, 1 deceased, 1 moved out of country, and 1 stopped using patch due to skin rash from cutaneous patch. More details were described in the main effects paper [21].

### Characteristics of the Study Participants

Among the 112 patients included in the final analysis, 53 (25 from the IS arm) completed the study before the pandemic (finished the last visit at week 28 before lockdown); and 59 (29 from the IS arm) had at least one study visit after the lockdown. Characteristics of patients by IS and UC group, and by before and after lockdown were reported in Table 1. There was no difference when comparing all characteristics between IS and UC groups at baseline (IS: n = 54, UC: n = 58). When comparing all characteristics between those who completed the study before lockdown and those who had at least one visit after lockdown, there were no differences in the UC arm. However, in the IS group, patients who had at least one visit completed after lockdown were younger than those who completed before lockdown (mean: 44 vs. 50 years, t = 2.07, p = 0.043).

### Missing Scheduled Study Visits

**Fig. 3:**
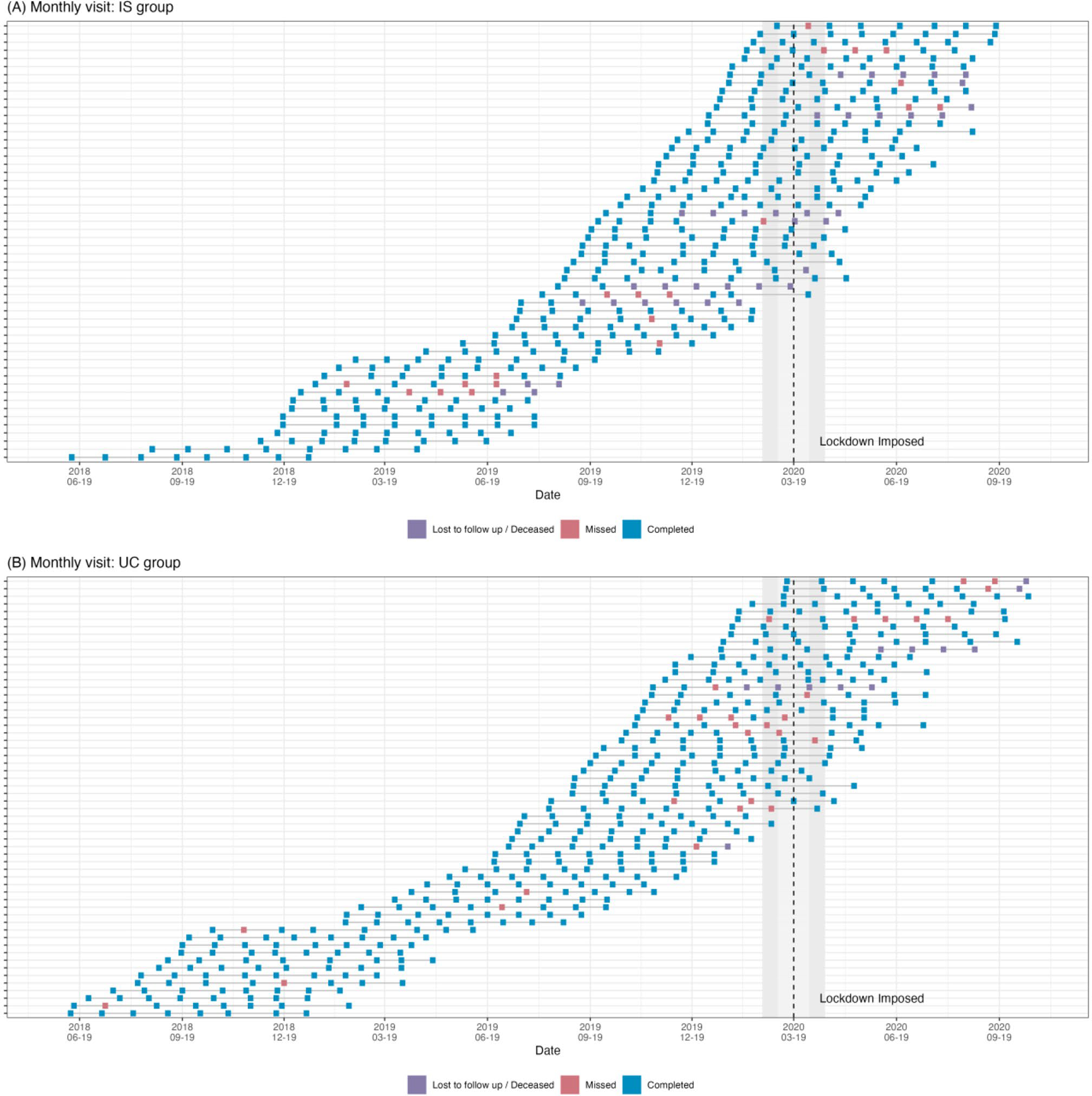
Profile of missing study visits for (**A**) ingestible sensor (IS) group; (**B**) usual care (UC) group Each row represented one participant in the study arm. Blue squares represented the completed study visits and red squares the missed study visits. Purple square indicates lost to follow up. The vertical dashed line on the graph depicts the time of the COVID-19 lockdown

All patients completed baseline assessments before the lockdown. Among the patients who were assigned to the IS group (n = 65), five patients withdrew from study; one of them was unable to complete the baseline visit due to COVID-19. One patient stopped using the IS system at week 4 due to COVID-19 concern and was followed without the IS system. For the UC group, one withdrew due to COVID-19. After baseline visits, seven participants were lost to follow-up because of COVID-19; five in the IS group and two in the UC group. One participant in the IS group went out of the country after week 8 and was stuck abroad due to COVID-19 until week 24. Another stopped wearing the patch from week 8 to 12 due to concerns about the adhesive patch and COVID-19. Among all participants, only one in the IS group reported having COVID-19, resulting the inability to attend one study visit. We profiled the study visits of both study groups (IS: n = 54, UC: n = 58) before and after COVID-19 lockdown in Fig. 3A and B. The profile plot visualized the overall study visits of two arms before and after lockdown. The missing and lost to follow up happened 2–4 weeks before the lockdown and continued after the lockdown time. From the profile, the evenly distributed blue squares indicated the COVID-19 pandemic did not have significant impact on our study. We did not find a significant increase in red squares (missing study visits) during and after the lockdown. Also, we did not find increased missing visits after intervention period among IS group, i.e., week 16–28.

### Adherence

**Fig. 4:**
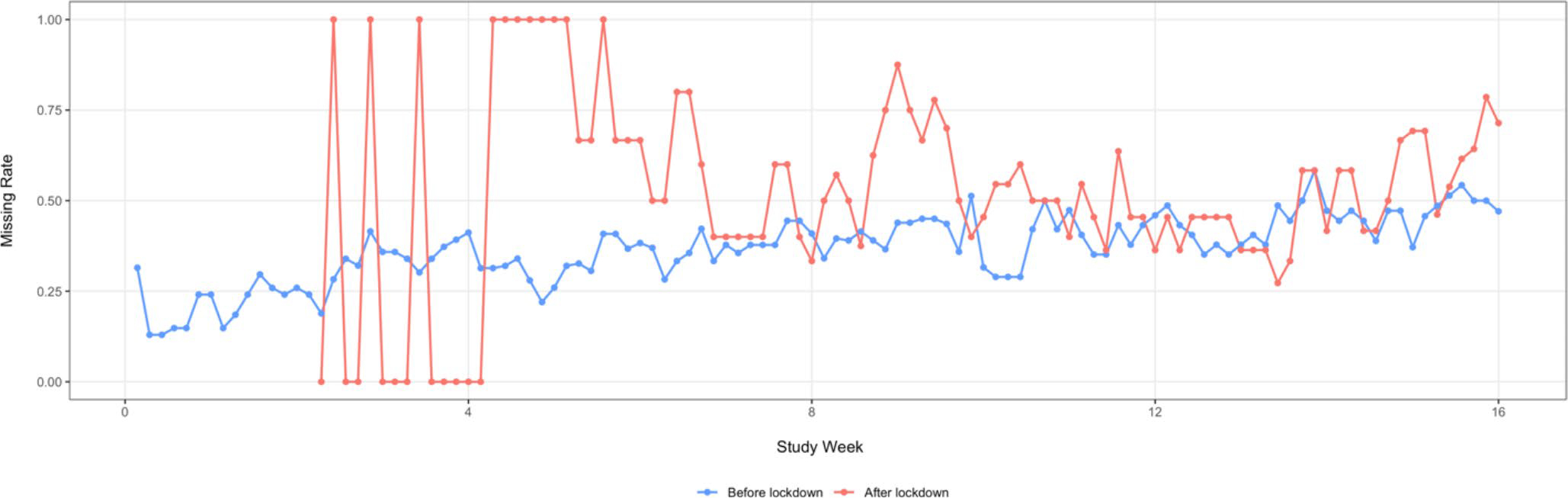
Compare Ingestion Sensor-monitored missing dose rate before and after COVID-19 lockdown

In the IS group (n = 54), daily real-time adherence measurements were available during the first 16 weeks. We compared the daily rate of missed doses before and after the lockdown in this group. Figure 4 presented the daily missing rate over intervention period. We found a significant difference in the IS measured adherence after week eight between those who finished the study before the lockdown (n = 25) and those who had at least one study visit happened after the lockdown (n = 29; t = −5.23, p < 0.001). In the first six weeks of the after-lockdown group, there was a large variation due to zero new enrollments. The last enrolled patient in the IS arm was in the third week of using the sensor at the time of the lockdown.

### Plasma HIV RNA

**Fig. 5:**
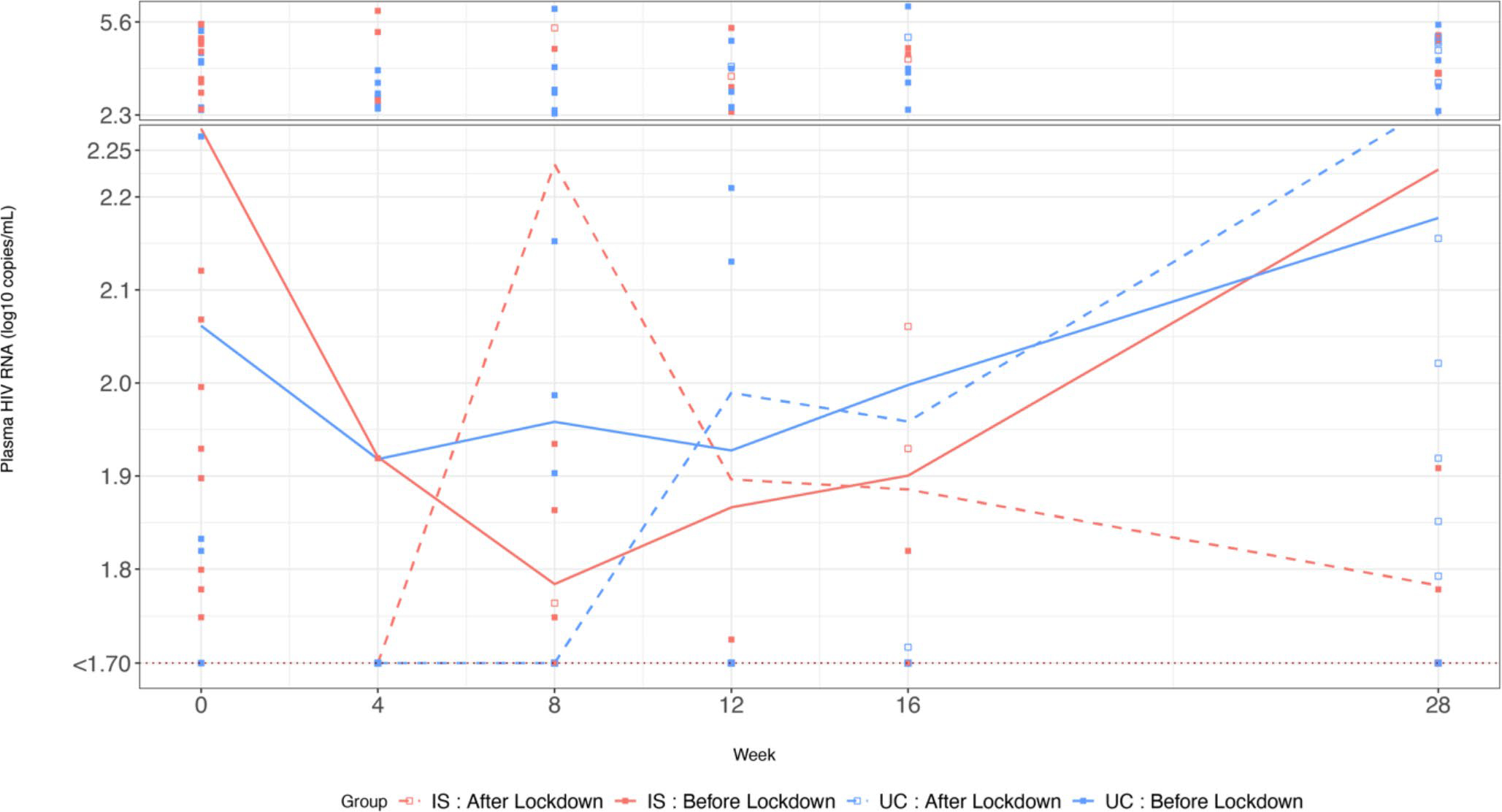
Compare plasma HIV RNA (log_10_ copies/mL before and after COVID-19 lockdown by ingestion sensor (IS) and Usual Care (UC) study arms Plasma HIV RNA was depicted in a solid line for the period before the lockdown and a dashed line for after the lockdown. In the scatter plot, we used filled-squares to represent before and empty squares for after the lockdown. We used the color red to represent the IS group and blue for the UC group

Figure 5 presents the HIV VL in log_10_ scale by four groups; the two study arms and by before and after lockdown. Overall, plasma HIV RNA measures between the IS and UC groups before the lockdown was not significant (n = 25, 28; t = −0.15, p = 0.89), although during the intervention period it decreased more in the IS group than the UC group. Notably, after the lockdown the IS group had a lower plasma HIV RNA than the UC group (n = 29, 30; t = 2.13, p = 0.04). For both before and after lockdown, we noticed the difference in plasma HIV RNA was greater in the IS than UC group, but not statistically significant (t = 1.52, −0.39; p = 0.15, 0.70).

### Study Visits and Plasma HIV RNA Suppression

In Table 2, we report the percentage of patients who had plasma HIV RNA suppressed. We report the number of participants in each two-by-two group. Before the lockdown, at week 28, UC group had higher percentage of participants who achieved viral suppression than IS group (80% vs. 70.6%). In contrast, after the lockdown, the percent with plasma HIV RNA suppression was lower in the UC than IS group at week 28 (61.5% vs. 96%); however, rates were similar at the end of intervention (week 16, 84.6% vs. 82.4%).

### Longitudinal Model

Table 3 summarizes the results of three RIAS models. All three models had the same outcome variable, longitudinal plasma HIV RNA (log_10_ scale) from baseline to week 28. The common independent variables in all three models included intervention group (IS: n = 54, UC: n = 58), study week with a quadratic term to reflect the non-linear relationship between plasma HIV RNA and study week in Fig. 5, self-reported adherence, before or after the COVID-19 lockdown, and their interactions. Model 1 was the simplest model including only the above common variables, indicating that the COVID-19 pandemic had some impact on the association between plasma HIV RNA and adherence (β = −0.81, p = 0.037). After lockdown, with a one-unit increase in adherence, HIV RNA decreased more, controlling for other variables. Model 2 suggested that there was no three-way interaction and the impact of the pandemic was not different between the IS and UC study groups. Model 3 adjusted for demographics, baseline characteristics, and behavior variables. Higher bimonthly self-reported adherence to ARVs was associated with lower plasma HIV RNA over time (β = −1.88, p = 0.004), after controlling for all covariates in Model 3. Furthermore, lockdown strengthened the associations between adherence and plasma HIV RNA (with interaction β = −2.27, p = 0.03). Before the lockdown, every 10% increase in adherence was associated with a 0.2 unit decrease in log_10_ plasma HIV RNA (p = 0.004), while after the lockdown occurred, the association was 0.41 units per log_10_ decrease in plasma HIV RNA (p = 0.03).

## Discussions

A major issue during the COVID-19 pandemic includes its potential impact on integrity of clinical trials that were impacted by local and regional lockdowns. As noted from Xue’s study in 2021, the number of clinical trials suspended due to COVID-19 increased rapidly from March to April 2020, and suspensions were released slowly in June 2020 [22]. In Los Angeles, from the first COVID-19 cases being reported in January 2020, to the lockdown order issued in March 2020, the design and implementation of randomized clinical trials have encountered more interruptions than traditional challenges, such as the impact on loss-to-follow up and biases of blindness [23]. Despite the pandemic, we, however, continued the study during the lockdown time. In our study, we used the IS system to monitor adherence for 16 weeks and during a 12-week post-intervention period prior to and after the COVID-19 lockdown. The system included the sensor pills co-encapsulated with the daily ARV, a patch on the abdominal wall that transferred a signal from the sensor to a smart device., The intervention also included a pre- and post-dose reminder text message if non-adherence events were detected. We have evaluated three main outcomes prior to and after the COVID-19 lockdown: missed study visits, adherence to medication, and plasma HIV RNA levels. After six-week of COVID-19 lockdown-related disruption, the daily rate of missed medication was close to those measured before the pandemic, though still slightly higher. This may be consistent with study guidelines from FDA that recommended that during the first few weeks of COVID-19 disruption, clinical trials should minimize study visits until the restrictions are lifted [13, 14]. We found the COVID-19 pandemic did not have an impact on our study visits and missed medication events in the IS group, but the plasma HIV RNA levels were lower amongst the participants in the IS than UC group. The plasma HIV RNA suppression rates were higher among participants in the IS (96.0% (95% CI: 78.6%, 100.0%)) than the UC group (61.5% (95%CI: 42.5%, 77.5%)) after the COVID-19 lockdown.

At the time of the COVID-19 lockdown, we began to record missed visits or study dropouts due to the COVID-19 pandemic or difficulties in site visits. During the lockdown, the clinic continued HIV study visits, so missing likely resulted from common factors, such as transportation issues, but may also have been impacted by COVID-19-related social distancing orders, and participant preference. For example, there were patients who cancelled or postponed the appointment or study visits. At the beginning of the first 4–8 weeks, we received 1 patient in the IS group who requested to stop wearing the patch due to concerns of the pandemic or transportation during the lockdown. Although an isolated event in our study, this is consistent with what has been reported in other clinical trials when comparing the number of patients enrolled in 2019 and 2020, 80% reported a decrease in enrollment [22]. There were a few other events that might have had a negative impact on the study due to COVID-19 related actions. For example, limited availability of public transportation often caused missed appointments or delayed study visits. We did not measure how many study visits were rescheduled or the duration of the delays. In our analysis, we used the categorical variable “study visit” in our model. We also had two participants who had the baseline evaluation before the lockdown but were then lost to follow up. And finally, we had another patient who requested to switch to the UC group after the lockdown. Since these patients only had baseline information, we did not include them in our analysis.

Few other studies have attempted to assess the impact of local COVID-19 pandemic lockdown on a clinical trial. This was particularly useful to make such an assessment since the study was designed to use innovative technology to systematically assess treatment adherence and how it relates to plasma HIV RNA levels. There were some limitations, including the fact that the COVID-19 pandemic and lockdown happened late towards the end of the study. During the lockdown, we offered the option for our patients to complete their study visits remotely. However, according to our records, no patients used this option. We did not recruit new patients due to the lockdown. Therefore, our study participants in both arms still completed all study visits in-person. These changes in the study that were instituted because of the COVID-19 pandemic and lockdown may have contributed to the relatively small impact. Hence the estimation of intervention effects may be biased if these factors were not considered. For people living with HIV, they are having new challenges besides adherence to medication and retention to HIV care. Anything happening during a clinical trial may have an impact on the results.

Many HIV clinics and research programs continued their services during the COVID-19 lockdown. It is well-recognized that we need to optimize the HIV care when resources are limited, especially at the time of a pandemic [9]. Some strategies were being implemented at the time of the lockdown, such as [24–28] (1) telehealth consultation and virtual appointment; (2) dispensing ARV for more than one month to reduce hospital or pharmacy visits; (3) postal express to deliver medications; (4) community-level health services; and (5) expansion of HIV self-testing and home health monitoring technologies. Similar strategies were adopted by many research protocols in order to maintain the integrity of ongoing studies during the lockdown. In conclusion, the impact of COVID-19 on patient care and the conduct of clinical trials poses new challenges to interpretation of trial results and its usefulness for future clinical practice. Further research is needed to develop innovative approaches that can better account for the impact of pandemic when analyzing study data.

## Figures and Tables

**Table 1:** Baseline characteristics of participants by ingestible sensor and usual care group and by before and after lockdown

Mean (SD; range) or N (%)	Ingestible Sensor (n = 54)		Usual Care (n = 58)	
	Before lockdown (n = 25)	After lockdown (n = 29)	Before lockdown (n = 28)	After lockdown (n = 30)

Age, years	50.0 (11.1; 31.8–65.5)	43.8 (10.6; 25.8–61.2)	46.1 (12.4; 26.9–75.1)	45.4 (12.6; 23.3–71.6)
Gender				
Female	2 (8.0%)	3 (10.3%)	3 (10.7%)	2 (6.7%)
Male	19 (76.0%)	25 (86.2%)	20 (71.4%)	23(76.7%)
Male to Female	4 (16.0%)	1 (3.4%)	5 (17.9%)	5 (16.7%)
Race/Ethnicity				
White	0 (0.0%)	6 (20.7%)	2 (7.1%)	6 (20.0%)
Black	14 (56.0%)	10 (34.5%)	16 (57.1%)	14 (46.7%)
Latino	9 (36.0%)	10 (34.5%)	9 (32.1%)	7 (23.3%)
Asian	1 (4.0%)	0 (0.0%)	1 (3.6%)	0 (0.0%)
Other	1 (4.0%)	3 (10.2%)	0 (0.0%)	3 (10.0%)
Education				
8th grade or less	3 (12.0%)	5 (17.2%)	5 (17.9%)	5 (16.7%)
High school	19 (76.0%)	19 (65.5%)	18 (64.3%)	21 (70.0%)
College and above	3 (12.0%)	5 (17.2%)	5 (17.9%)	4 (13.3%)
Employment				
Part-time/full-time	3 (12.0%)	6 (20.7%)	9 (32.1%)	10 (33.3%)
Not working	22 (88.0%)	23 (79.3%)	19 (67.9%)	20 (66.7%)
HIV + Years, years	16.6 (7.9; 4–29)	12.7 (7.8; 0–32)	12.4 (8.5;1–33)	15.4 (9.8; 0–34)
Years under ARV Treatment, years	14.3 (7.3; 4–28)	11.0 (7.4; 0–27)	10.6 (7.6; 1–29)	12.5 (7.8; 0–28)
History of AIDS Diagnosis				
Yes	3 (12.0%)	9 (31.0%)	7 (25.0%)	5 (16.7%)
No	20 (80.0%)	19 (65.5%)	21 (75.0%)	23 (76.7%)
Unknown	2 (8.0%)	1 (3.4%)	0 (0.0%)	2 (6.7%)
Detectable VL at Baseline				
<= 50 copies/mL	15 (60.0%)	20 (69.0%)	20 (71.4%)	26 (86.7%)
> 50 copies/mL	10 (40.0%)	9 (31.0%)	8 (28.6%)	4 (13.3%)
Plasma HIV RNA at Baseline, log_10_ copies/mL *	2.4 (1.2; 1.7–5.0)	2.2 (1.2; 1.7–5.5)	2.3 (1.2; 1.7–5.5)	1.9 (0.7; 1.7–5.3)

ARV, antiretroviral; SD, standard deviation; VL, viral load

* For patients with undetectable HIV RNA plasma, viral load was all treated as 50 copies/mL

**Table 2:** Number and percentage of participants with plasma HIV RNA < 50 copies/mL before and after COVID-19 lockdown by study arm

Week	Usual Care		Ingestible Sensor	
	Before lockdown	After lockdown	Before lockdown	After lockdown

0	58 (46, 79.3%)	0	54 (35, 64.8%)	0
4	49 (41, 83.7%)	3 (3, 100%)	51 (45, 88.2%)	1 (1, 100%)
8	46 (37, 80.4%)	9 (9, 100%)	40 (36, 90.0%)	7 (5, 71.4%)
12	42 (35, 83.3%)	8 (7, 87.5%)	36 (32, 88.9%)	10 (9, 90.0%)
16	38 (33, 86.8%)	13 (11, 84.6%)	29 (26, 89.7%)	17 (14, 82.4%)
28	25 (20, 80.0%)	26 (16, 61.5%)	17 (12, 70.6%)	25 (24, 96.0%)

Number with data available (number < 50 copies/mL, percent < 50 copies/mL)

**Table 3:** Longitudinal mixed effects models with random intercept and slope (RIAS) on plasma HIV RNA change before and after COVID-19 lockdown

	Variables	Coefficient ± Std error	p-value

Model 1	Intercept	2.373 ± 0.196	< 0.0001
	Intervention (IS)	0.084 ± 0.107	NS
	Study week	−0.030 ± 0.009	0.0010
	Study week^2^	0.001 ± 0.0003	0.0002
	Adherence (ADH)	−0.385 ± 0.197	0.0508
	Lockdown	0.734 ± 0.358	0.0407
	Lockdown x ADH	−0.810 ± 0.386	0.0365
Model 2	Intercept	2.359 ± 0.197	< 0.0001
	Intervention (IS)	0.107 ± 0.112	NS
	Study week	−0.029 ± 0.009	0.0011
	Study week^2^	0.001 ± 0.0003	0.0002
	ADH	−0.383 ± 0.197	0.0521
	Lockdown	0.732 ± 0.358	0.0415
	Lockdown x ADH	−0.732 ± 0.401	NS
	IS x Lockdown x ADH	−0.138 ± 0.176	NS
Model 3	Intercept	5.321 ± 1.678	0.002
	Intervention (IS)	−0.039 ± 0.247	NS
	Study week	−0.006 ± 0.062	NS
	Study week^2^	0.001 ± 0.002	NS
	Bimonthly ADH	−1.875 ± 0.644	0.004
	Lockdown	1.740 ± 0.938	NS
	Lockdown x Bimonthly ADH	−2.273 ± 1.029	0.028
	Age	−0.027 ± 0.014	NS
	Race		
	Caucasian	Ref.	Ref.
	Asian/Pacific	−0.535 ± 1.313	NS
	African American	−0.039 ± 0.432	NS
	Latinx/Hispanic	0.150 ± 0.443	NS
	Other	−0.077 ± 0.608	NS
	Education		
	8th grade or less	Ref.	Ref.
	High school	0.373 ± 0.381	NS
	College and above	0.207 ± 0.494	NS
	Employment		
	Part-time/full-time	Ref.	Ref.
	Not working	0.738 ± 0.304	0.018
	Multiple regimen	1.235 ± 1.270	NS
	VL detectable at baseline	1.284 ± 0.303	< 0.0001
	CD4 count at baseline	0.002 ± 0.001	0.033
	Years on ARV	0.008 ± 0.021	NS
	Bimonthly CD4	−0.003 ± 0.001	< 0.0001
	Depression	0.600 ± 0.262	0.025
	Illicit drug use	0.135 ± 0.282	NS

NS – not significant (> 0.05)

ADH, adherence; ARV, antiretroviral; IS, ingestible sensor; VL, viral load
